# Comparison of Efficacy of the Disease-Specific LOX1- and Constitutive Cytomegalovirus-Promoters in Expressing Interleukin 10 through Adeno-Associated Virus 2/8 Delivery in Atherosclerotic Mice

**DOI:** 10.1371/journal.pone.0094665

**Published:** 2014-04-15

**Authors:** Hongqing Zhu, Maohua Cao, Leonardo Mirandola, Jose A. Figueroa, Everardo Cobos, Maurizio Chiriva-Internati, Paul L. Hermonat

**Affiliations:** 1 Medical Research Service, Central Arkansas Veterans Healthcare System, Little Rock, Arkansas, United States of America; 2 Department of Internal Medicine, Division of Hematology & Oncology, Texas Tech University, Health Sciences Center, School of Medicine, Lubbock, Texas, United States of America; 3 Kiromic LLC, Lubbock, Texas, United States of America; Medical University of Graz, Austria

## Abstract

The development of gene therapy vectors for treating diseases of the cardiovascular system continues at a steady pace. Moreover, in the field of gene therapy the utility of “disease-specific promoters” has strong appeal. Many therapeutic genes, including transforming growth factor beta 1 or interleukin 10, are associated to adverse effects. The use of a disease-specific promoter might minimize toxicity. The lectin-like oxidized low density lipoprotein receptor 1 is a marker of cardiovascular disease and a potential therapeutic target. The lectin-like oxidized low density lipoprotein receptor 1 is known to be up-regulated early during disease onset in a number of cell types at the sites where the disease will be clinically evident. In this study an adeno-associated virus-2 DNA vector (AAV2) using the AAV8 capsid, and containing the full length The lectin-like oxidized low density lipoprotein receptor 1 promoter, was generated and assayed for its ability to express human interleukin 10 in low density lipoprotein receptor knockout mice on high cholesterol diet. The cytomegalovirus early promoter was used for comparison in a similarly structured vector. The two promoters were found to have equal efficacy in reducing atherogenesis as measured by aortic systolic blood velocity, aortic cross sectional area, and aortic wall thickness. This is the first head-to-head comparison of a constitutive with a disease-specific promoter in a therapeutic context. These data strongly suggest that the use of a disease-specific promoter is appropriate for therapeutic gene delivery.

## Introduction

Atherosclerosis can be described as an inflammation of arteries [1], [2]. Cells of the monocyte/macrophage/foam cell lineage are numerically prominent during plaque development, and may represent the cell type with the highest etiologic basis for atherogenesis [3], [4]. Anti-inflammatory cytokines such as transforming growth factor beta 1 (TGFβ1) or interleukin 10 (IL10) might be used for therapeutic effect, but these agents have significant adverse reactions [5], [6]. For example the phenotype of TGFβ1 is very pleiomorphic and is highly associated with the induction of fibrosis [7]–[10]. In comparison, IL10 has fewer complications yet is still associated with a number of adverse reactions such as increased infections, anemia, and headache [11]–[20]. We consider that gene therapy is the most advantageous way to deliver IL10 because of its short half-life. Adeno-associated virus (AAV) has been known to be an effective vector for gene therapy since its first use in 1984 [21]–[23], and continues to increase in popularity [24]–[26]. Three different reports have described successful AAV-delivered IL10 resulting in the inhibition of atherogenesis in mouse models [27]–[29]. Moreover, the ability of AAV to deliver therapeutic genes for up to 10 years in patients [30] should ultimately be advantageous in making gene therapy cost effective as well as improving the quality of life of patients.

Due to the adverse effects of using IL10 therapeutically, the use of a disease-specific promoter to control its expression in the sites of arteriosclerosis would likely be beneficial. The use of such disease-specific promoters has been modestly studied for the expression of marker genes [31], [32]. LOX1 encodes for a scavenger receptor which binds oxidized low density lipoprotein (Ox-LDL) and is expressed by activated endothelial cells, smooth muscle cells, and macrophages, the major cell types believed involved in atherosclerosis. Thus it is highly expressed within the atherosclerotic plaque [33]–[37]. LOX-1 is also considered one of the earliest markers of activated endothelium, an event which precedes the atherosclerosis process itself [33]. LOX1 appears to be transcriptionally up-regulated during atherogenesis and many agents, such as Ox-LDL and AngII, induce this up-regulation. Therefore, LOX1 transcriptional promoter (LOX1pr) may be a useful disease-specific promoter to express therapeutic genes to counteract atherogenesis. Here we compare AAV2/8.LOX1pr-hIL10 gene delivery with that of previously tested AAV2/8.CMVpr-hIL10 and we show that both promoters provide statistically similar efficacy against atherosclerosis in an *in vivo* mouse model.

## Materials and Methods

### Generation of Recombinant AAV Virus

We used the full length 2.4 kb LOX1 promoter previously characterized [33]–[40]. To generate the AAV/LOX1pr-IL10, the full length Lox1 promoter (nt −2402 to +9) was amplified from human HEK-293 cell by PCR using the primers: upstream 5′-AT*ATGCAT*CTTTCTTATTTGGGGGAAG-3′ and downstream 5′-AT*ACGCGT*ACTAAAAATATGTGAGCTTCTG-3′. *Nsi* I and *Mlu* I sites (underlined) were included in the primers to allow easy ligation upstream of the hIL10 gene within the AAV2 plasmid dl3–97. Construction and generation of AAV/Neo and AAV/CMVpr-hIL10 recombinant virus have been described previously [27], [41]. We used the cytomegalovirus immediate early constitutive promoter (CMVpr) for comparison against the LOX1pr, which is well studied in gene therapy [42], [43]. The virus stocks were generated and titrated by dot blot hybridization as described previously [27], [41]. The titers of different virus preparations were brought to 1×10^9^ encapsidated genomes per mL (eg/mL).

### Animal Treatments

The low density lipoprotein receptor (LDLR) knockout mouse strain (B6; 129S7-*Ldlr^tm1Her^*/J) was purchased from Jackson Laboratories (Bar Harbor, ME, USA). Two groups of male mice (n = 8–10) weighing 16–20 grams were injected with AAV2/8.LOX1pr-hIL10, AAV2/8.CMV-hIL10, or AAV2/8.SV40pr-Neo virus (2×10^8^ eg/mouse in 200 µL). each at a titer of 1x 10^9 ^eg/mL via tail vein injections of 200 µL virus/mouse, followed by two booster injections at an interval of approximately 5 days. High cholesterol diet (HCD), consisting of 4% cholesterol and 10% Coco butter (Harlan Teklad, Madison, WI, USA), was provided on the day of first injection and continuously maintained for twenty weeks. HCD was used to ensure the development of atherosclerosis. Negative control mice were fed normally. All experimental procedures were performed in accordance with protocols approved by the Institutional Animal Care and Usage Committee of the Central Arkansas Veterans Health Care System at Little Rock. All animals were weighed weekly starting at 16 weeks post-injection.

### High Resolution Ultrasound Imaging

The Vevo 770 High-Resolution Imaging system (Visualsonics, Toronto, Canada) was used for ultrasound imaging, using an RMV 707B transducer, having a center frequency of 30 MHz. Animals were prepared as described earlier [41]. Briefly, eight to ten mice from each group were anesthetized with 1.5% isoflurane (Isothesia, Abbot Laboratories, Chicago, USA), with supplemental oxygen and laid supine on a thermostatically heated platform. Legs were taped to ECG electrodes for cardiac function monitoring. A shaver was used to remove abdominal hair along with a chemical hair remover (Church & Dwight Co, Inc., NJ, USA) and pre-warmed US gel (Medline Industries, Inc., Mundelein, USA) was then spread over the skin as a coupling medium for the transducer. The thoracic/abdominal region of the aorta was visualized, below the aortic arches to the diaphragm. Image acquisition was started in the B-mode, where a long axis view was used to visualize the length of the aorta. The scan probe was then turned 90° for a short-axis view to obtain pictures of the cross-sectional area of the aorta. Individual frames and cine loops (300 frames) were also acquired at all levels of the aorta both in long axis and short axis view and recorded at distances of 1 mm throughout the length of the aorta. The flow velocity, orientation of the abdominal aorta on ultrasound, was accomplished by tilting the platform and the head of mouse down with the transducer probe towards the feet and tail of the mouse. The described positioning ensured that the Doppler angle was less than 60° for accurate measurements of blood flow velocity in the pulse-wave Doppler (PW) mode within the aorta. Measurements and data analysis was performed off-line on the longitudinal and transverse images using the customized version of Vevo770 Analytical Software. It took approximately 25–30 minutes to carry out the complete imaging for each mouse.

### Measurement of Plasma Cholesterol

The Veterans Animal Laboratory (VAMU) determined the plasma levels of total cholesterol for the animal groups, measured by VetScan VS2 (ABAXIS, Union City, CA).

### hIL10 Gene Expression Analysis Using Real-time Quantitative Reverse Transcription PCR (QRT-PCR)

Six mice from each group were sacrificed and total aortic RNA was extracted with TRIzol extraction (Invitrogen Carlsbad, CA) according to the manufacturer’s instructions. cDNA was generated using random hexamer primers and RNase H-reverse transcriptase (Invitrogen, Carlsbad, CA). QRT-PCR was then performed using the Applied Biosystems Fast 7900HT real-time PCR system (Applied Biosystems, Foster City, CA) as described [41]. We designed qRT-PCR specific primers for analyzing human IL10 using Probe-Finder (roche-applied-science.com) web-based software from Roche Applied Science. The results were further analyzed using SDS 2.3 relative quantification (RQ) manager software. The comparative threshold cycles (Ct) values were normalized for the βactin reference gene and then compared with a calibrator by the 2^−ΔΔCt^ method.

### Statistics

IL-10, body weight, cholesterol and the hemodynamic parameters (aortic systolic blood velocity, aortic cross sectional area, and aortic wall thickness) were analyzed with statistics software SPSS 16.0 by nonparametric ANOVA test. If differences were detected between means, Newman-Keuls test was used for multiple comparisons. Difference were considered as significant if *P*<0.05.”

## Results

### AAV2/8 Delivers hIL10

**Figure 1:**
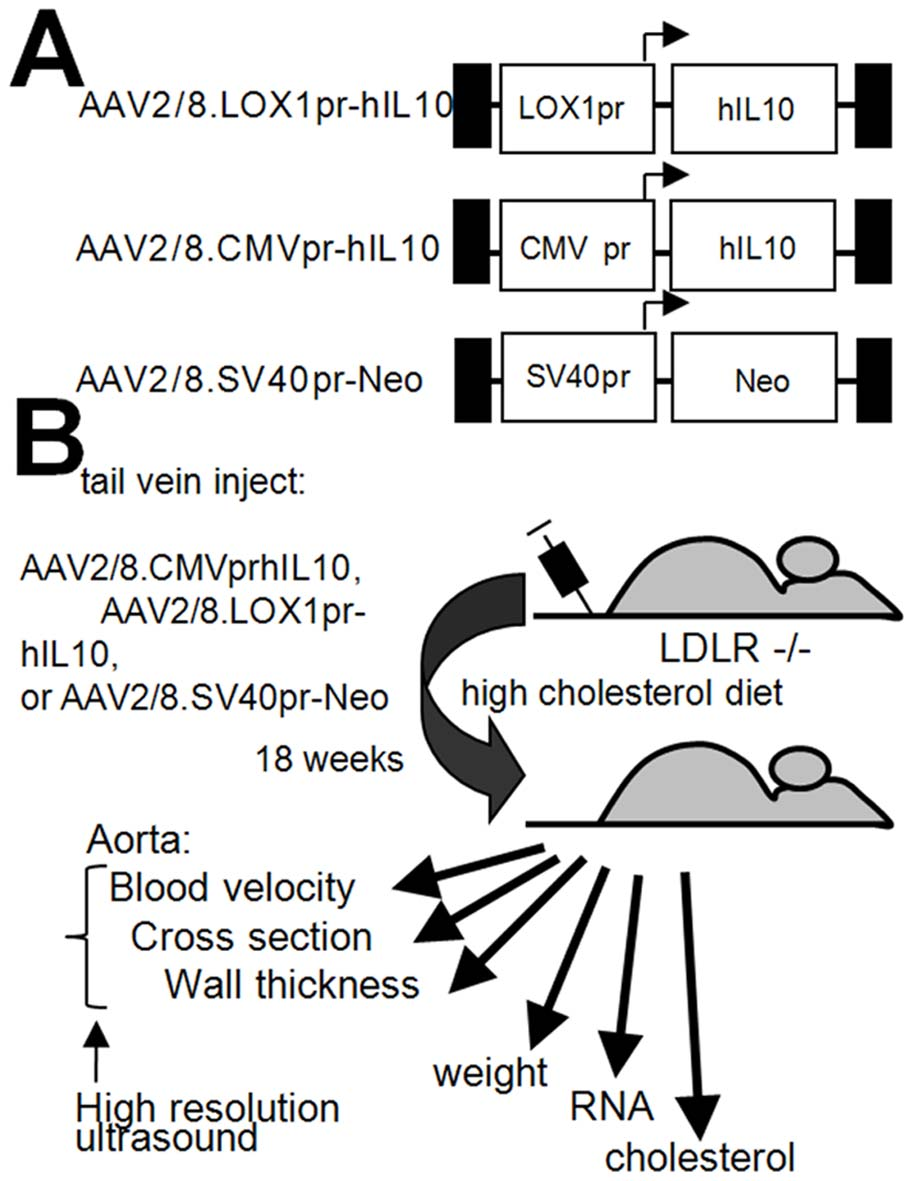
Structure of AAV vectors and experimental scheme. **A.** Basic structure of the three AAV vectors used in this study. **B.** Experimental scheme and measurements.

**Figure 2:**
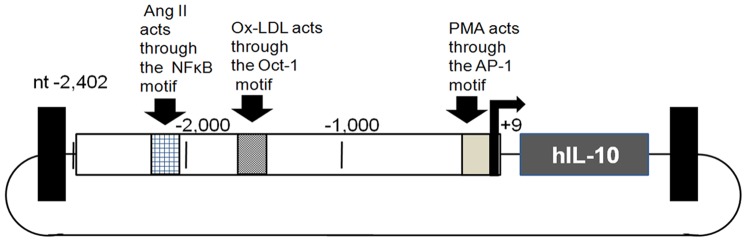
Structure of the AAV.LOX1pr-hIL10 vector plasmid. This figure depicts some of the general sites that are known to operate within the human LOX1pr within human endothelial cells or cardiomyocytes.

**Figure 3:**
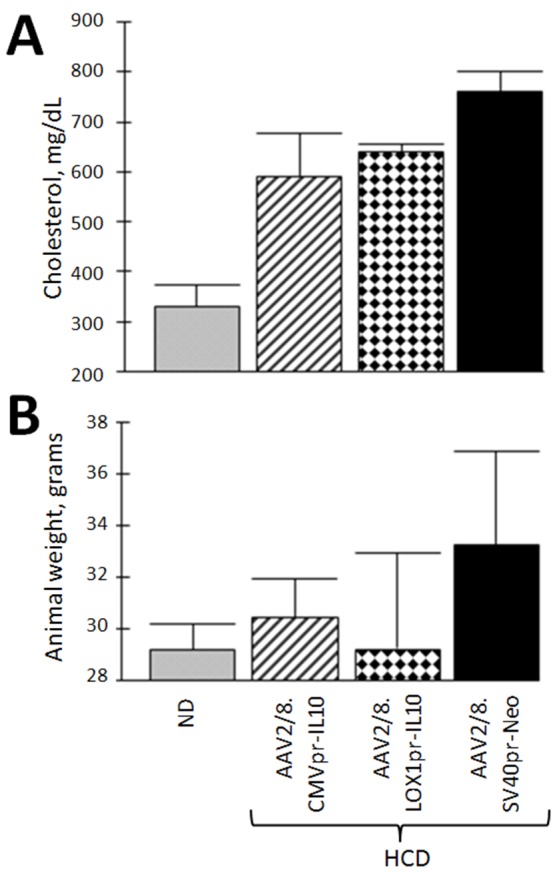
Cholesterol levels and animal weight. Data shown are mean +/− SE. **A.** shows the levels of total cholesterol (*p*<0.05). **B.** shows the animal weights at the end of the experiment. There is no significant weight different among groups (*p* = 0.140). The key at the bottom is used for both panels.

We consider IL10 as a “gold standard” anti-atherosclerotic gene as it has been used and shown to be efficacious by three different groups [27]–[29]. However, before moving on to clinical trials we considered that it may be appropriate to investigate the use of a disease-specific promoter to limit the possibility of adverse reactions to systemic IL-10 expression. To test this hypothesis, the efficacy AAV2/8.LOX1pr-IL10 was compared to AAV2/8.CMVpr-IL10 in LDLR KO mice on high cholesterol diet (HCD). An AAV/Neomycin resistance gene (Neo) vector (AAV2/8.SV40pr-Neo) was also used as a non-therapeutic, null control. Vector structures are shown in Figure 1A and the overall experimental scheme in Figure 1B. A more detailed view of the AAV.LOX1pr-hIL10 vector plasmid is shown in Figure 2. 20 weeks after virus administration, 6 mice from each group were sacrificed, and mRNA isolated from the aortas used to measure the expression of IL10. Representative results for LOX1pr- and CMV-hIL10–treated mice are shown in Figure 3, and both vectors were observed to be highly expressed in aortas at a level of appropriately 0.1–0.2% that of βactin. Results showed that the expression driven by by CMVpr and LOX1pr were not statistically different.

### Both CMVpr-hIL10 and LOX1pr-hIL10 Transgene Delivery Inhibit Aortic Blood Flow Velocity with Equal Efficacy

**Figure 4:**
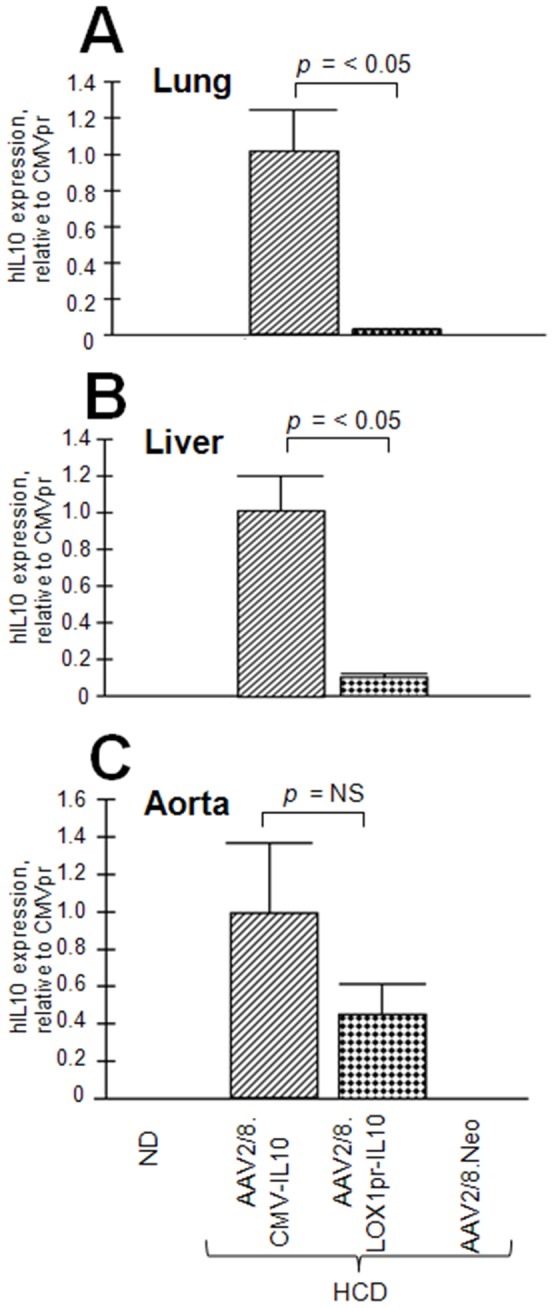
Expression of hIL10 in animal groups. Relative expression of delivered hIL10 determined by quantitative PCR from the aortas of 6 mice per group. Data shown are mean +/− SE. Note that high expression of hIL10 by LOX1pr-hIL10-HCD-treated animals took place only in the aortas.

Having demonstrated significant gene delivery/expression for both CMVpr-hIL10 orLOX1pr-hIL10, we then studied the effects of the transgene. Figure 4A shows that CMVpr-hIL10 and LOX1pr-hIL10-transgene-HCD-treated animals had similar total cholesterol levels, and were comparable to Neo-treated, HCD-fed animals, however hIL-10 treated animals trended slightly lower as has been seen previously. No significant changes of animal weight were observed in the different groups (Figure 4B).

**Figure 5:**
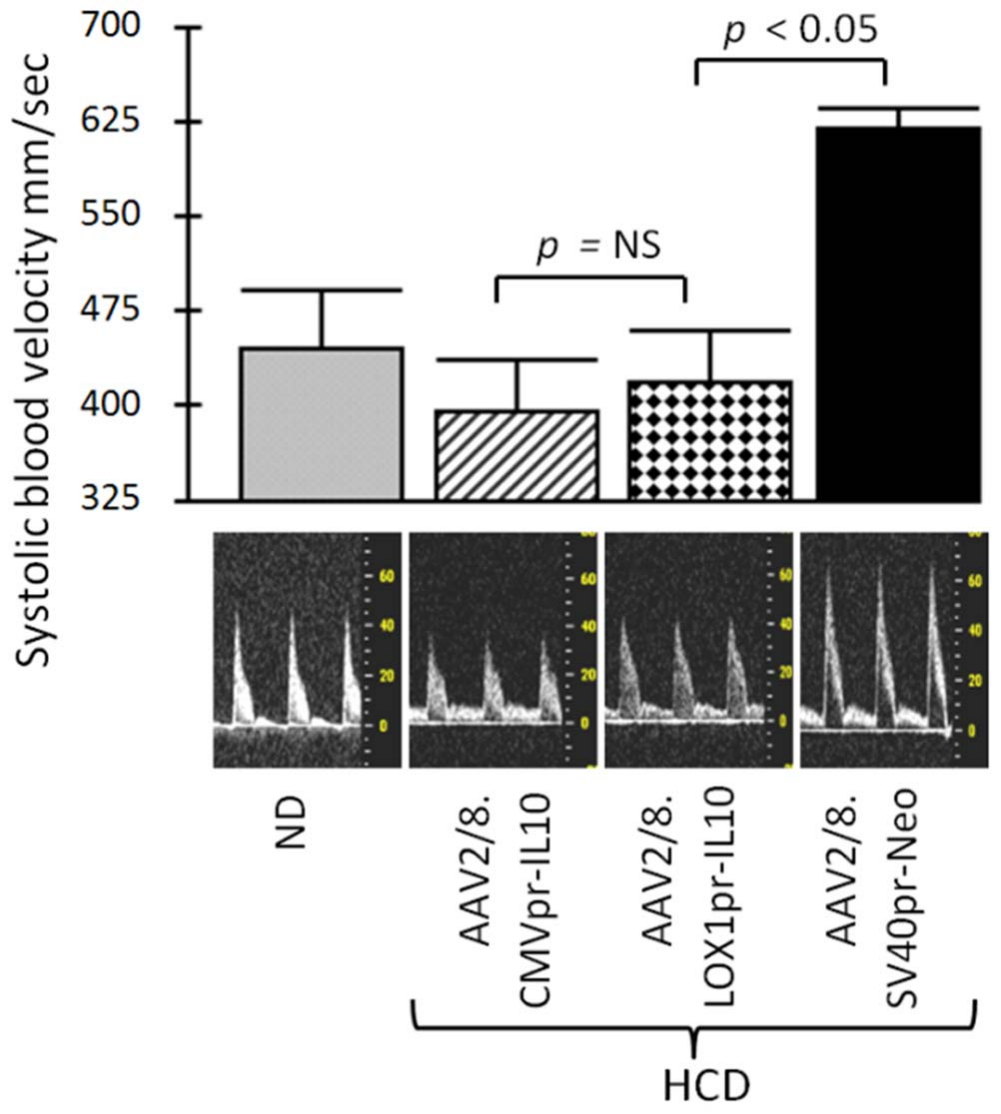
Systolic blood velocity. High resolution ultrasound (HRUS) was used to measure blood flow velocities in the luminal center of the abdominal region of the aorta in 8–10 animals from each group. Note that both CMVpr-hIL10- and LOX1pr-hIL10-HCD-treated animals had significantly lower blood velocity than the AAV/Neo-HCD-treated animals, similar to ND controls (*p*<0.05). However, CMVpr-hIL10-HCD- and LOX1pr-hIL10-HCD-treated animals were statistically similar to each other (*p* = 0.377).

We analyzed arterial blood flow velocity as an easy technique to calculate the extent of atherosclerosis in the mouse groups. The systolic blood velocity in the thoracic/abdominal region of the aorta was quantified by high resolution ultrasound (HRUS) imaging system Vevo 770 with measurements taken on eight-to-ten animals. Figure 5 shows the systolic blood velocity from five separate measurements on each animal. Neo-HCD-treated animals displayed significantly higher aorta blood flow velocities (*p*<0.05), consistent with a constricted aortic lumen. Additionally, both CMVpr- and LOX1pr- driven gene therapies (hIL-10) on HCD had markedly lower flow velocity than the Neo-HCD-treated group, and very similar to that of the ND fed control animals (*p*<0.05). Thus, as suggested by lower blood velocity, both CMVpr- and LOX1pr- treatments displayed an anti-atherosclerotic effect and were statistically similar in their degree efficacy (*p* = 0.377).

### CMVpr-hIL10 and LOX1pr-hIL10 Gene Delivery Inhibits Aortic Structural Changes Associated with Atherosclerosis with Equal Efficacy

**Figure 6:**
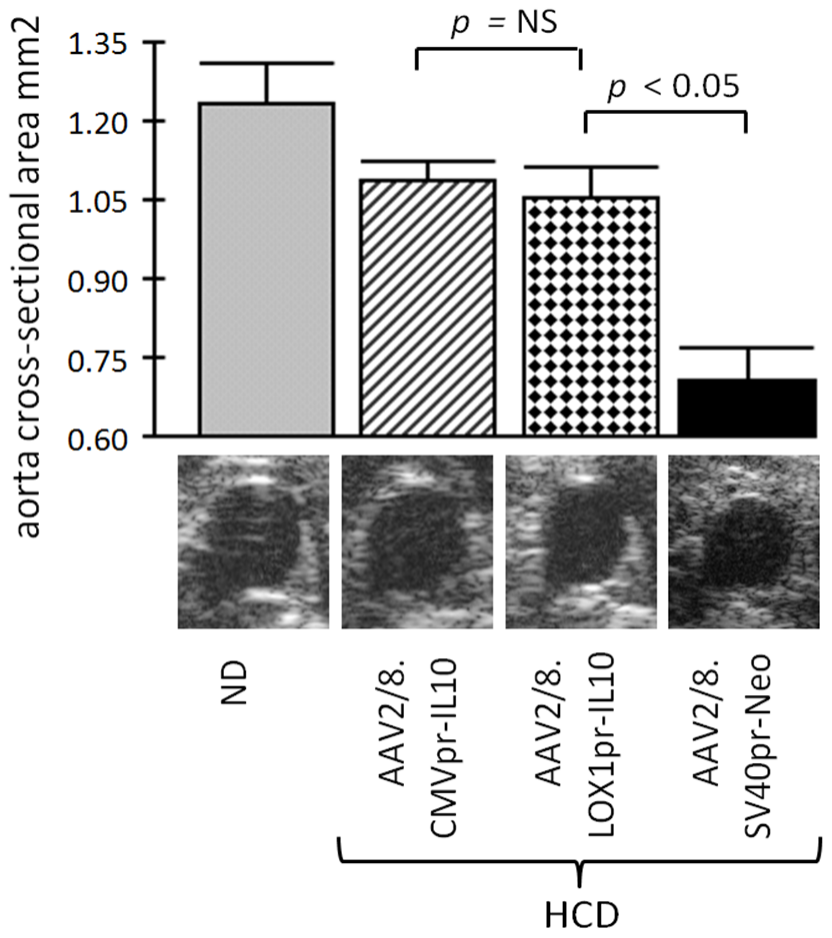
Analysis of the aortic lumen by high resolution ultrasound (HRUS). HRUS was used to measure the cross sectional area of the thoracic region of the aortas in 8–10 animals from each animal group. Shown is a quantification of the cross-sectional area for the abdominal/thoracic region of the aorta. Note that both CMVpr-hIL10- and LOX1pr-hIL10-HCD-treated animals had a significantly larger cross sectional area than the AAV/Neo-HCD-treated animals, indicating significant efficacy (*p*<0.05). However, CMVpr-hIL10-HCD- and LOX1pr-hIL10-HCD-treated animals were statistically similar to each other (*p* = 0.235).

We then analyzed structural changes within the aortas of the various experimental groups by measuring the aortic cross sectional area. Multiple measurements were taken from eight-to-ten animals in each group. The measurements were made in the same thoracic/abdominal site (see Materials and Methods). Figure 6 shows the results of the thoracic/abdominal region of the aorta. The AAV/Neo-HCD-treated had the smallest cross sectional lumen area, being consistent with atherosclerosis (*p*<0.05). The two therapeutic treatments, both LOX1pr-hIL10-HCD and CMVpr-IL10, had significantly larger lumens than Neo-treated-HCD controls (*p*<0.05).

**Figure 7:**
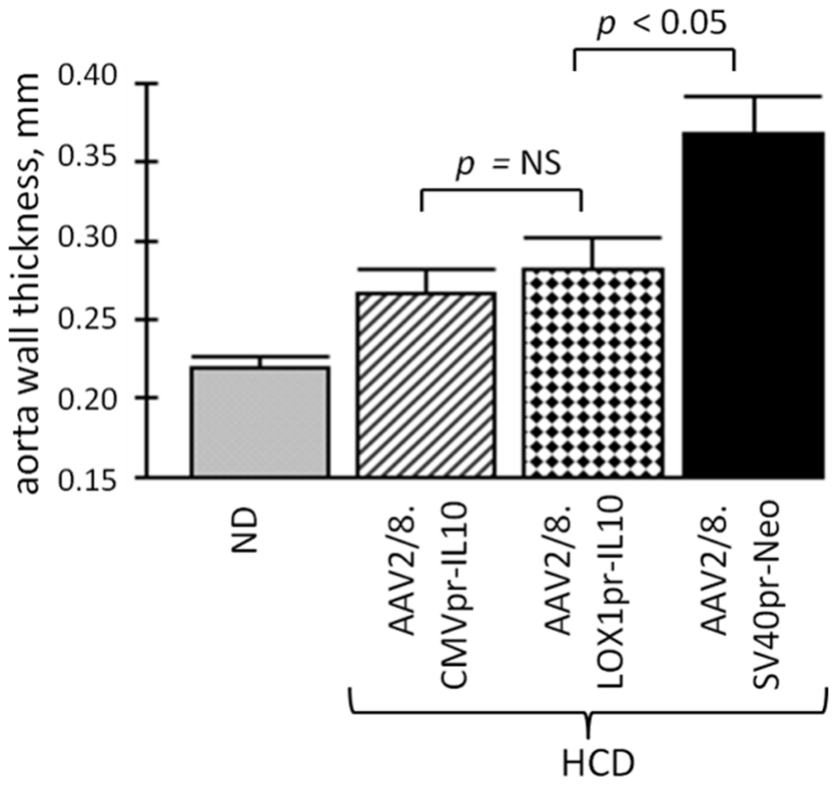
Analysis of the aortic wall thickness by HRUS. Shown is a quantification of the thoracic region of the aortas in 8–10 animals from each animal group. Note that both CMVpr-hIL10- and LOX1pr-hIL10-HCD-treated animals had a significantly thinner aortic wall than the AAV/Neo-HCD-treated animals (*p*<0.05), indicating significant efficacy. However, CMVpr-hIL10-HCD- and LOX1pr-hIL10-HCD-treated animals were statistically similar to each other (*p* = 0.144).

The wall thickness of the thoracic region of the aorta was a final measurement made on the aortas by HRUS. Figure 7 shows the results for the thoracic region of the aorta. The Neo-HCD-treated animals displayed the thickest aortic walls, while both CMVpr- and LOX1pr-hIL10 vector-HCD-treatments were efficacious (*p*<0.05) and were statistically similar to each other (*p* = 0.144). The normal diet group had the thinnest aortic walls.

## Discussion

Here we demonstrate that an AAV2/8.LOX1pr-hIL10 vector was effective in inhibiting atherogenesis in LDLR KO mice on HCD, and that its efficacy was equal to that of the AAV2/8.CMV-IL10 vector. Additionally, there were no deaths or observed morbidity as a result of IL10 delivery by either AAV vector, even though IL10 is linked to higher infection rates [11]. We believe this is the first head-to-head comparison of a disease-specific promoter (LOX1) and a well used constitutive promoter (CMV) in an *in vivo* setting. The cytomegalovirus immediate early promoter is perhaps the most used promoter in gene delivery experiments. Thus this study shows that disease-specific promoters, when chosen appropriately, are still able to express therapeutic transgenes to efficacious levels. Experiments to identify the specificity of LOX1pr-IL10 expression within the atherosclerotic plaque were not successful, not unexpectedly, as IL10 is a secreted product. Yet there is enough documentation that the 2.4 kb LOX1 promoter fragment has a significant activity and responsiveness to disease-associated stimuli as does the wt LOX-1 promoter [38]–[40]. The basal level of LOX1 promoter activity is very low [39]. Although we did not determine what stimulus was responsible for LOX1pr induction in our animal model, it is likely that Ox-LDL plays a role, but other stimulations are also possible, such as that of angiotensin II [44]. One additional question is why LOX1pr-IL10 with HCD is not expressed very highly in the lung and liver which also have significant arterial mass (endothelial and smooth muscle cells)? Although we did not quantitate the fat content it was visually apparent that the livers were fatty and the lungs were not. In the liver, AAV8 readily transduces hepatocytes and likely these cells become a major reservoir for AAV8 infection, thereby competitively reducing the level of transduction of liver arteries [45]. It is also interesting that fatty livers appear associated with atherosclerosis at other sites but not in the liver itself [46]. Endothelial cells are not a major target for AAV8 [47].

While LOX-1 is the “gold standard” as an atherogenesis-active promoter, there are other genes that are similarly up-regulated [48]–[52]. Also, the milieu of cells present within the artery wall during atherogenesis changes over time [53], [54], and this should be taken into account when choosing the disease-specific promoter for gene delivery. In normal arteries low monocyte populations exist within the arterial wall, while over time, with the activation of the endothelium and recruitment of additional monocytes, this number dramatically increases. Yet, our AAV vectors are systemically delivered before HCD-initiated disease. Thus, the transduction of the whole monocyte pool is likely, part of which will ultimately migrate into the arterial wall. Alternatively, Robbins et al (2013) suggest that resident intra-arterial monocyte populations are major contributors to monocyte/macrophage/foam cell burdens [55]. Yet, even then, AAV-delivered IL10 expression within the arterial walls should limit the proliferation of this lineage [56]. The most appropriate mouse/diet model to best study plaque formation is still a matter of debate. The LDLR KO mouse does not develop atherosclerosis without a HCD. Yet, the level of cholesterol (4%) and coco butter (10%) in HCD is very high, perhaps more than in some human Western diets.

We have previously described the therapeutic use of AAV/IL10 and its downstream effector, STAT3, to limit atherogenesis in the LDLR KO mouse. This series of studies [27], [57], [58], along with those of others [28], [29], have shown that IL10, and the IL10 signal transduction pathway, is beneficial for limiting atherogenesis by gene therapy. Our first study, with IL10 being expressed from AAV2 p5 promoter, demonstrated that IL10 was effective in inhibiting plaque development in the LDLR KO mouse on HCD [27]. We have also demonstrated that STAT3, a downstream mediator of IL10 signaling, confers anti-atherogenesis effect [57]. In any case, this study represents a significant step forward in the debate on usefulness of disease-specific promoters for gene therapy of arteriosclerosis disease. This study adds to the weight of many previous studies and further shows the suitability and versatility of adeno-associated virus vectors for cardiovascular gene therapy.

## References

[1] Libby P, Ridker PM, Maseri A (2002) Inflammation and Atherosclerosis. Circulation. 105: 1135–1143. 10.1161/hc0902.104353 11877368

[2] Libby P, Ridker PM, Hansson GK (2009) Inflammation in atherosclerosis. J Amer Coll Card 54: 2129–2138. 10.1016/j.jacc.2009.09.009 PMC2834169 19942084

[3] Ludewig B, Laman JD (2004) The in and out of monocytes in atherosclerotic plaques: Balancing inflammation through migration. Proc Natl Acad Sci U S A. 101: 11529–11530. 10.1073/pnas.0404612101 PMC511015 15292506

[4] Woollard KJ, Geissmann F (2010) Monocytes in atherosclerosis: subsets and functions. Nat Rev Cardiol. 7: 77–86. 10.1038/nrcardio.2009.228 PMC2813241 20065951

[5] Letterio JJ, Roberts AB (1998) Regulation of immune responses by TGF-beta. Annu Rev Immunol 16: 137–161. 9597127 10.1146/annurev.immunol.16.1.137

[6] de Vries JE (1995) Immunosuppressive and anti-inflammatory properties of interleukin 10. Ann Med. 27: 537–41. 10.3109/07853899509002465 8541028

[7] Byfield SD, Roberts AB (2004) Lateral signaling enhances TGF-beta response complexity. Trends Cell Biol 14: 107–111. 15055198 10.1016/j.tcb.2004.01.001

[8] Attisano L, Wrana JL (2002) Signal Transduction by the TGF-beta Superfamily. Science 296: 1646–1647. 12040180 10.1126/science.1071809

[9] Kopp JB, Factor VM, Mozes M, Nagy P, Sanderson N, et al (1996) Transgenic mice with increased plasma levels of TGF-beta 1 develop progressive renal disease. Lab Invest 74: 991–1003. 8667617

[10] Gressner AM, Weiskirchen R, Breitkopf K, Dooley S (2002) Roles of TGF-beta in hepatic fibrosis. Front Biosci 7: 793–807. 10.2741/A812 11897555

[11] Filippi CM, von Herrath MG (2008) IL-10 and the resolution of infections. High levels of IL10 are associated with increased viral, bacterial, and fungal infections, as well as cancer. J Pathol 214: 224–230. 18161757 10.1002/path.2272

[12] Zobel K, Martus P, Pletz MW, Ewig S, Prediger M, et al (2012) Interleukin 6, lipopolysaccharide-binding protein and interleukin 10 in the prediction of risk and etiologic patterns in patients with community-acquired pneumonia: results from the German competence network CAPNETZ. BMC Pulmonary Medicine. 12: 6. 10.1186/1471-2466-12-6 PMC3311562 22348735

[13] Clemons KV, Grunig G, Sobel RA, Mirels LF, Rennick DM, et al (2000) Role of IL-10 in invasive aspergillosis: increased resistance of IL-10 gene knockout mice to lethal systemic aspergillosis. Clin Exper Imm 122: 186–91. 10.1046/j.1365-2249.2000.01382.x PMC1905763 11091273

[14] Brooks DG, Lee AM, Elsaesser H, McGavern DB, Oldstone MB (2008) IL-10 blockade facilitates DNA vaccine-induced T cell responses and enhances clearance of persistent virus infection. J Exp Med 205: 533–541. 18332180 10.1084/jem.20071948 PMC2275377

[15] Brooks DG, Trifilo MJ, Edelmann KH, Teyton L, McGavern DB, et al (2006) Interleukin-10 determines viral clearance or persistence in vivo. Nat Med 12: 1301–1309. 17041596 10.1038/nm1492 PMC2535582

[16] Ejrnaes M, Filippi CM, Martinic MM, Ling EM, Togher LM, et al (2006) Resolution of a chronic viral infection after interleukin-10 receptor blockade. J Exp Med 203: 2461–2472. 17030951 10.1084/jem.20061462 PMC2118120

[17] Zeni E, Mazzetti L, Miotto D, Lo Cascio N, Maestrelli P, et al (2007) Macrophage expression of interleukin-10 is a prognostic factoir in nonsmall cell lung cancer. Eur Respir J 30: 627–632. 17537769 10.1183/09031936.00129306

[18] Maris CH, Chappell CP, Jacob J (2007) Interleukin-10 plays an early role in generating virus-specific T cell anergy. BMC Immunol 8: 8. 17570849 10.1186/1471-2172-8-8 PMC1903364

[19] Herfarth H, Schölmerich J (2002) IL-10 therapy in Crohn’s disease: at the crossroads Gut. 50: 146–147. 10.1136/gut.50.2.146 PMC1773119 11788549

[20] Asadullah K, Sterry W, Volk HD (2003) Interleukin-10 Therapy–Review of a New Approach Pharmacological Reviews. 55: 241–269. 10.1124/pr.55.2.4 12773629

[21] Hermonat PL, Muzyczka N (1984) Use of adeno-associated virus as a mammalian DNA cloning vector: transduction of neomycin resistance into mammalian tissue culture cells. Proc Natl Acad Sci USA 81: 6466–6470. 6093102 10.1073/pnas.81.20.6466 PMC391945

[22] Tratschin JD, West MH, Sandbank T, Carter BJ (1984) A human parvovirus, adeno-associated virus, as a eukaryotic vector: transient expression and encapsidation of the prokaryotic gene for chloramphenicol acetyltransferase, Mol Cell Biol. 4: 2072–2081. 10.1128/mcb.4.10.2072-2081.1984 PMC369024 6095038

[23] Hermonat PL, Labow MA, Wright R, Berns KI, Muzyczka N (1984) Genetics of adeno-associated virus: isolation and preliminary characterization of mutants in adeno-associated virus type 2. J Virol 51: 329–339. 6086948 10.1128/jvi.51.2.329-339.1984 PMC254442

[24] Liu Y, Chiriva-Internati M, Grizzi F, Salati E, Roman JJ, et al (2001) Rapid induction of cytotoxic T cell response against cervical cancer cells by human papillomavirus type 16 E6 antigen gene delivery into human dendritic cells by an adeno-associated virus vector. Can Gene Ther 8: 948–957. 10.1038/sj.cgt.7700391 11781657

[25] Chiriva-Internati M, Liu Y, Weidanz JA, Grizzi F, You H, et al (2003) Testing recombinant adeno-associated virus-gene loading of dendritic cells for generating potent cytotoxic T lymphocytes against a prototype self-antigen, multiple myeloma HM1.24. Blood. 102: 3100–3107. 10.1182/blood-2002-11-3580 12855576

[26] You CX, Shi M, Liu Y, Cao M, Luo RC, et al (2012) AAV2/IL-12 gene delivery into dendritic cells (DC) enhances CTL stimulation above other IL-12 applications: evidence for IL-12 intracrine activity in DC. Oncoimm 2012 1: 847–855. 10.4161/onci.20504 PMC3489740 23162752

[27] Liu Y, Li D, Chen J, Xie J, Bandyopadhyay S, et al (2006) Inhibition of atherogenesis in LDLR knockout mice by systemic delivery of adeno-associated virus type 2-hIL-10. Atherosclerosis 188: 19–27. 16300768 10.1016/j.atherosclerosis.2005.10.029

[28] Chen S, Kapturczak MH, Wasserfall C, Glushakova OY, Campbell-Thompson M, et al (2006) Interleukin 10 attenuates neointimal proliferation and inflammation in aortic allografts by a heme oxygenase-dependent pathway. Proc Natl Acad Sci 102: 7251–7256. 10.1073/pnas.0502407102 PMC1090475 15878989

[29] Yoshioka T, Okada T, Maeda Y, Ikeda U, Shimpo M, et al (2004) Adeno-associated virus vector-mediated interleukin-10 gene transfer inhibits atherosclerosis in apolipoprotein E-deficient mice. Gene Therapy 11: 1772–1779. 15496963 10.1038/sj.gt.3302348

[30] Jiang H, Pierce GF, Ozelo MC, de Paula EV, Vargas JA, et al (2006) Evidence of multiyear factor IX expression by AAV-mediated gene transfer to skeletal muscle in an individual with severe hemophilia B. Molec Ther. 24: 452–455. 10.1016/j.ymthe.2006.05.004 16822719

[31] Wettergren EE, Gussing F, Quintino L, Lundberg C (2012) Novel disease-specific promoters for use in gene therapy for Parkinson’s disease. Neurosci Lett 530: 29–34. 23063686 10.1016/j.neulet.2012.09.059

[32] Kim HA, Mahato RI, Lee M (2009) Hypoxia-specific gene expression for ischemic disease gene therapy Adv Drug Del Rev. 61: 614–622. 10.1016/j.addr.2009.04.009 19394379

[33] Sawamura T, Kume N, Aoyama T, Morlaki H, Hoshikawa H, et al (1997) An endothelial receptor for oxidized low-density lipoprotein. Nature 386: 73–77. 9052782 10.1038/386073a0

[34] Oka K, Sawamura T, Kikuta K, Itokawa S, Kume N, et al (1998) Lectin-like oxidized low density lipoprotein receptor 1 mediates phagocytosis of aged/apoptotic cells in endothelial cells. Proc Natl Acad Sci USA 95: 9535–9540. 9689115 10.1073/pnas.95.16.9535 PMC21373

[35] Mehta JL, Li D (1998) Identification and autoregulation of receptor for OX-LDL in cultured human coronary artery endothelial cells. Biochem Biophys Res Commun 248: 511–514. 9703956 10.1006/bbrc.1998.9004

[36] Kakutani M, Masaki T, Sawamura T (2000) A platelet-endothelium interaction mediated by lectin-like oxidized low-density lipoprotein receptor-1. Proc Natl Acad Sci USA. 97: 360–364. 10.1073/pnas.97.1.360 PMC26668 10618423

[37] Ogura S, Kakino A, Sato Y, Fujita Y, Iwamoto S, et al (2009) LOX-1: the multifunctional receptor underlying cardiovascular dysfunction. Circ J 73: 1993–1999. 19801851 10.1253/circj.cj-09-0587

[38] Aoyama T, Sawamura T, Furutani Y, Matsuoka R, Yoshida MC, et al (1999) Structure and chromosomal assignment of the human lectin-like oxidized low-density-lipoprotein receptor-1 (LOX-1) gene. Biochem J. 339: 177–184. PMC1220142 10085242

[39] Chen J, Liu Y, Liu H, Hermonat PL, Mehta JL (2006) Lectin-like oxidized low-density lipoprotein receptor-1 (LOX-1) transcriptional regulation by Oct-1 in human endothelial cells: implications for atherosclerosis. Biochemical J 393: 255–265. 10.1042/BJ20050845 PMC1383684 16173915

[40] Chen J, Liu Y, Liu H, Hermonat PL, Mehta JL (2006) Molecular dissection of angiotensin II-activated human LOX-1 promoter. Arterioscler Thromb Vasc Biol. 26: 1163–1168. 10.1161/01.ATV.0000209998.73303.b5 16484599

[41] Khan JA, Cao M, Kang BY, Liu Y, Mehta JL, et al (2010) Systemic hNetrin-1 gene delivery lowers monocyte/macrophage accumulation and atherogenesis *in vivo* . Gene Therapy 18: 437–444. 21160531 10.1038/gt.2010.155

[42] Qin JY, Zhang L, Clift KL, Hulur I, Xiang AP, et al (2010) Systematic comparison of constitutive promoters and the doxycyline-inducible promoter. PLOSone 5: e10611. 10.1371/journal.pone.0010611 PMC2868906 20485554

[43] Zarrin AA, Malkin L, Fong I, Luk KD, Ghose A, et al (1999) Comparison of CMV, RSV, SV40 viral and V lambda 1 cellular promoters in B and T lymphoid and non-lymphoid cell lines. Biochim Biophys Acta 1446: 135–139. 10395926 10.1016/s0167-4781(99)00067-6

[44] Hermonat PL, Zhu H, Cao M, Mehta JL (2011) LOX-1 transcription. Cardiovasc Drugs Therapy. 25: 393–400. 10.1007/s10557-011-6322-8 21796333

[45] Brea A, Mosquera D, Martin E, Arizti A, Cordero JL, et al (2005) Nonalcoholic fatty liver disease is associated with carotid atherosclerosis: a case– control study. Arterioscler Thromb Vasc Biol 25: 1045–1050. 15731489 10.1161/01.ATV.0000160613.57985.18

[46] Grimm D, Pandey K, Nakai H, Storm TA, Kay MA (2006) Liver transduction with recombinant adeno-associated virus is primarily restricted by capsid serotype not vector genotype. J Virol 80: 426–439. 16352567 10.1128/JVI.80.1.426-439.2006 PMC1317553

[47] Denby L, Nicklin SA, Baker AH (2005) Adeno-associated virus (AAV)-7 and -8 poorly transduce vascular endothelial cells and are sensitive to proteasomal degradation. Gene Ther 12: 1534–1538. 15944729 10.1038/sj.gt.3302564

[48] Faber BC, Cleutjens KB, Niessen RL, Aarts PL, Boon W, et al (2001) Identification of genes potentially involved in rupture of human atherosclerotic plaques. Circ Res 89: 547–554. 11557743 10.1161/hh1801.096340

[49] Armstrong PJ, Johanning JM, Calton Jr WC, Delatore JR, Franklin DP, et al (2002) Differential gene expression in human abdominal aorta: aneurysmal versus occlusive disease. J Vasc Surg 35: 346–355. 11854734 10.1067/mva.2002.121071

[50] Hiltunen MO, Tuomisto TT, Niemi M, Brasen JH, Rissanen TT, et al (2002) Changes in gene expression in atherosclerotic plaques analyzed using DNA array. Atherosclerosis 165: 23–32. 12208467 10.1016/s0021-9150(02)00187-9

[51] Martinet W, Schrijvers DM, De Meyer GR, Thielemans J, Knaapen MW, et al (2002) Gene expression profiling of apoptosis-related genes in human atherosclerosis: upregulation of death-associated protein kinase. Arterioscler Thromb Vasc Biol 22: 2023–2029. 12482829 10.1161/01.atv.0000041843.44312.12

[52] Woodside KJ, Hernandez A, Smith FW, Xue XY, Hu M, et al (2003) Differential gene expression in primary and recurrent carotid stenosis. Biochem Biophys Res Commun 302: 509–514. 12615063 10.1016/s0006-291x(03)00191-8

[53] Stary HC (2000) Natural History and Histological Classification of Atherosclerotic Lesions: An Update Arterioscler Thromb Vasc Biol. 20: 1177–1178. 10.1161/01.atv.20.5.1177 10807728

[54] Stone GW, Maehara A, Lansky AJ, de Bruyne B, Cristea E, et al (2011) A prospective natural-history study of coronary atherosclerosis. N Engl J Med 364: 226–235. 21247313 10.1056/NEJMoa1002358

[55] Robbins CS, Hilgendorf I, Weber GF, Theurl I, IwamotoY, et al (2013) Local proliferation dominates lesional macrophage accumulation in atherosclerosis. Nat Med 19: 1166–1172. 23933982 10.1038/nm.3258 PMC3769444

[56] O’Farrell AM, Liu Y, Moore KW, Mui AL (1998) IL-10 inhibits macrophage activation and proliferation by distinct signaling mechanisms: evidence for Stat3-dependent and -independent pathways. EMBO J 17: 1006–18. 9463379 10.1093/emboj/17.4.1006 PMC1170450

[57] Khan JA, Cao M, Kang BY, Mehta JL, Hermonat PL (2010) AAV/hSTAT3-gene delivery lowers aortic inflammatory cell infiltration in LDLR KO mice on high cholesterol diet. Atherosclerosis 213: 59–66. 20727521 10.1016/j.atherosclerosis.2010.07.029

[58] Cao M, Khan JA, Kang BY, Mehta JL, Hermonat PL (2012) Dual AAV/IL-10 plus STAT3 anti-inflammatory gene delivery lowers atherosclerosis in LDLR KO mice, but without increased benefit. Int J Vasc Med. 2012: 524235. 10.1155/2012/524235 PMC3170890 21915378

